# Elevated Platelet Aggregation in Patients with Ovarian Cancer: More than Just Increased Platelet Count

**DOI:** 10.3390/cancers16213583

**Published:** 2024-10-24

**Authors:** Zitha Redempta Isingizwe, Brooke A. Meelheim, Doris Mangiaracina Benbrook

**Affiliations:** 1 Department of Pharmaceutical Sciences, College of Pharmacy, University of Oklahoma Health Sciences Center, Oklahoma City, OK 73117, USA; zitha-isingizwe@ouhsc.edu; 2 Division of Gynecologic Oncology, Department of Obstetrics and Gynecology, Stephenson Cancer Center, University of Oklahoma Health Sciences Center, Oklahoma City, OK 73104, USA; meelheim@gmail.com

**Keywords:** ovarian cancer, platelet aggregation, aggregation slope, thrombocytosis, thrombosis

## Abstract

**Background:** Patients with ovarian cancer have high platelet counts, which correlate with disease burden, incidence, and lethality of blood clots (thrombosis). We hypothesized that elevated aggregation is associated with both increased platelet number and altered behavior of platelets in patients with ovarian cancer. **Methods:** Healthy controls and patients with suspected or diagnosed ovarian cancer were evaluated for complete blood counts. To evaluate the effects of platelet count versus platelet behavior, equal platelet-rich plasma (PRP) volumes versus equal platelet numbers were used in platelet aggregation assays. Arachidonic acid, adenosine diphosphate, and collagen platelet agonists were used to induce aggregation. Volunteers were grouped into healthy controls (23), benign/borderline cases (7), and cancer cases (25 ovarian, 1 colorectal, and 2 endometrial). **Results:** The rate and amount of platelet aggregation were higher in patients compared to healthy controls regardless of whether the same platelet number or PRP volume was used. Compared to healthy controls, patients with untreated ovarian cancer exhibited high levels of platelet activation markers, P-selectin (27.06 vs. 31.06 ng/mL, *p* = 0.03), and beta-thromboglobulin (3.073 vs. 4.091 µg/mL, *p* = 0.02) in their plasma. The significance of the elevation and its correlations with platelet number or PRP volume varied depending on the agonist. Platelet (305.88 vs. 134.12, *p* < 0.0001) and white blood cell (8.459 vs. 5.395, *p* < 0.01) counts (×10^9^/L) were elevated pre-chemotherapy and decreased post-chemotherapy, respectively. **Conclusions:** Elevated platelet aggregation is caused by both altered platelet number and behavior in patients with ovarian cancer. These results support the study of antiplatelet agents for thrombosis prevention in these patients.

## Simple Summary

Patients with ovarian cancer have elevated platelet counts and an increased risk of dying from blood clots. It is logical that this increased blood clot risk is caused by the increased numbers of platelets. However, to develop prevention for thrombosis in patients with ovarian cancer, it is important to know if the platelets in patients with ovarian cancer are different from the platelets in healthy individuals. In this study, we demonstrated through carefully controlled experiments that increased platelet aggregation, which is necessary for clotting, is caused by both the elevated numbers and altered behavior of platelets in patients with ovarian cancer. Furthermore, we show that the platelets from patients with ovarian cancer have elevated levels of platelet activation markers compared to healthy patients. Future experiments will evaluate why platelets in patients with ovarian cancer are more prone to clotting and will study medications that could reduce the increased blood clot risk.

## 1. Introduction

While cancer itself can be lethal, thrombosis is the second leading cause of death in patients with cancer [1]. Thromboembolisms interfere with chemotherapy treatment and patient quality of life, as well as resource utilization and costs to the healthcare industry [2,3]. Specifically, in ovarian cancer, thromboembolisms usually occur within the first two months of surgery, and prophylaxis of thromboembolisms has been shown to improve patient survival and decrease costs to the healthcare system [4]. Thus, an improved understanding of the cause of thromboembolism in patients with ovarian cancer could be used to develop a strategy for reducing lethality in patients with ovarian cancer. One theory that could explain the increased thromboembolism occurring in patients with ovarian cancer is the abnormally high platelet count (thrombocytosis) observed in up to 22–30% of newly diagnosed patients with ovarian cancer [5]. Thrombocytosis in patients with ovarian cancer is associated with poor prognosis, advanced disease stage, reduced progression-free survival (PFS), and increased risk of death compared to patients without thrombocytosis [5,6,7]. In vitro, in vivo, ex vivo and clinical data suggest that platelets promote ovarian cancer progression by activating various signaling pathways including intereleukin-6 (IL-6), nuclear factor kappa B and vascular endothelial growth factor pathways, among others [7]. Platelet targeting agents, such as antiplatelet antibodies or aspirin, reduced tumor growth and aggressiveness in male gastric cancer models [8], suggesting that platelets contribute to overall cancer aggressiveness in addition to thromboembolisms. In ovarian cancer mouse models, silencing of thrombopoietin and IL-6 reduced levels of thrombocytosis, suggesting that thrombopoietin and IL-6 are the major drivers of thrombocytosis in ovarian cancer [6]. In the same study, an IL-6 neutralizing antibody enhanced the cytotoxicity effect of paclitaxel in mice [6], which further supports the need to target platelets in ovarian cancer to improve patients’ prognosis.

Platelets also play a critical role in ovarian cancer chemoresistance. Clinical studies evaluating the association between thrombocytosis and ovarian cancer survival revealed that thrombocytosis at the time of diagnosis is associated with low PFS and reduced overall survival [9]. The same study also revealed that docetaxel efficacy in ovarian mouse models is enhanced by the addition of platelet-depleting antibodies while platelet transfusion abrogated the drug efficacy [9].

Khorana score, which uses physical parameters such as the type of cancer, the patient’s platelet and leukocyte counts, hemoglobin level, and body mass index (BMI) at the time of diagnosis is used to predict thrombosis risk in patients with cancer [10]. Khorana scoring has failed to place patients in the correct risk group on multiple occasions, as shown in a systematic review [11], leading to improper management of the disease state. This suggests that there might be additional key players affecting thrombosis risk such as the nature of platelet behavior and levels of circulating factors that regulate platelet aggregation.

Platelet aggregation studies comparing specimens from patients with ovarian cancer to those from healthy and/or benign controls have reported inconsistent results. One study comparing platelets from patients with ovarian cancer with a group of healthy and benign controls observed a higher percentage of ovarian cancer specimens (75%) exhibiting hyperactive platelets, defined as ≥30% aggregation induced by 2.5 μM ADP [12], compared to control specimens (27%) [13]. The same study found no significant difference in aggregation between the two groups when 10 μM ADP was used as the agonist, suggesting that platelets from ovarian cancer patients were not pre-activated. A subsequent study by the same group confirmed that there was no difference between specimens from patients with ovarian cancer and benign tumors regarding platelet hyper- or pre-activation induced by ADP or collagen [14]. Furthermore, this study found no difference in platelet pre-activation using platelet-poor plasma (PPP) levels of β-thromboglobulin (beta-TG) and platelet factor 4 (PF4) as biomarkers [14,15]. In the current study, we aimed to evaluate the contributions of different platelet aggregation agonists and elevated platelet numbers to the increased platelet aggregation in patients with ovarian cancer plasma. We evaluated complete blood count (CBC), including platelet size as a surrogate of platelet age, as well as cancer-associated platelet behavior and elevated platelet count effects on ex vivo agonist-induced aggregation of platelets from healthy controls, patients with benign tumors or borderline diseases, and patients with ovarian cancer. We hypothesized that elevated platelet aggregation in the plasma of patients with ovarian cancer is caused by both an elevated platelet count and altered platelet behavior.

## 2. Materials and Methods

### 2.1. Participant Recruitment

All studies were approved by the Institutional Review Board of the University of Oklahoma Health Sciences Center (IRB #1426). A total of 97 participants were recruited, screened, and provided informed consent to participate in this study. The eligibility criteria for healthy controls were as follows: female sex, age ≥ 42 years (42 is the youngest age in our patients with ovarian cancer group), medication history of no aspirin use within the last 14 days, and lack of previous history of any form of malignancy. Of the 47 volunteers that were screened, 23 participants were eligible based on their lack of history of cancer and not taking blood thinners at the time of the blood draw and were therefore included in this study. Seventeen healthy participants were excluded from the analysis because they were younger than 42, while seven healthy controls were excluded because they were unable to provide sufficient blood for this study, were found to be ineligible, or did not show up for the blood draw after being enrolled in this study. The eligibility criteria for patients with ovarian cancer were as follows: female sex, suspected epithelial ovarian cancer, or active diagnosis of epithelial ovarian cancer before scheduled cytoreductive surgery. The diagnosis of the surgical specimen as cancer or benign/borderline was confirmed by a gynecologic oncologist (B.A.M.) based on each patient’s pathology report. Twenty-eight patients with epithelial ovarian tumors were included in this study. Three cancer patients were excluded because they had germline or stromal tumors, two patients with cancer were excluded because they had recent cytoreductive surgery, and ten patients with cancer were excluded because they had other types of primary cancer with no involvement of the ovaries, were found to be ineligible, or did not show up for the blood draw after being enrolled in this study.

### 2.2. Blood Collection and Processing

Venous blood was directly collected in acid citrate dextrose (ACD, BD, Franklin Lakes, NJ, USA, 364606) anticoagulant. Five hundred microliters were used to determine the CBC using a VetScan HM5 Hematology Analyzer (Zoetis, Parsippany, NJ, USA) in the primate species function. Centrifugation (<300× *g*, 10 min, room temperature without brakes) was used to separate the platelet-rich plasma (PRP), the upper fraction, from red blood cells and other blood cells, which are the lower and middle fractions, respectively. The platelet count in the PRP was determined using a VetScan HM5 Hematology Analyzer (Zoetis). Platelet-poor plasma (PPP) control was collected from the upper fraction after blood centrifugation (~700× *g*, 10 min, room temperature).

### 2.3. Agonist-Induced Platelet Aggregation

A Platelet Aggregation Profiler (PAP-8E, Bio/Data Corporation, Horsham, PA, USA) was used to perform all platelet aggregation assays in a final volume of 250 µL. Low-dose of adenosine diphosphate (ADP, 2 µM final concentration Bio/Data Corporation, 101312) platelet agonist was used to induce partial aggregation, while high-dose of arachidonic acid (AA, 0.5 mg/mL final concentration, Bio/Data Corporation, 101297), adenosine diphosphate (20 µM final concentration Bio/Data Corporation, 101312) or collagen (Col, 0.19 mg/mL, Bio/Data Corporation, 101562) were used to induce full platelet aggregation. Differences in platelet behavior between healthy controls and patients with neoplasm diseases were evaluated by comparing aggregation patterns when an equal platelet number per assay (10 million platelets in 1:4 PPP in phosphate-buffered saline) was used. The direct effect of thrombocytosis was compared between the groups by evaluating equal volumes of PRP (25 µL PRP in 1:4 PPP in phosphate-buffered saline) per assay.

### 2.4. P-Selectin, PF4, and Beta-TG Human Luminex Discovery Assays

The Human Luminex Discovery Assay for P-Selectin/CD62P (Biotechne, R&D Systems, Horsham, PA, USA, LXSAHM-01) or CXCL4/PF4 and CXCL7/NAP-2 for beta-TG (Biotechne, R&D Systems, LXSAHM-02) was performed according to the manufacturer’s instructions. Briefly, plasma from frozen PRP was centrifuged at 16000× *g* prior to incubation with a microparticle cocktail. The samples were then incubated with a biotin-antibody cocktail followed by streptavidin-PE. A Bio-Plex 200 System (BioRad, Hercules, CA, USA) was used to collect median fluorescence intensities based on the region of each analyte.

### 2.5. Data Analysis and Statistical Test

Prism 10.2 software (GraphPad, La Jolla, CA, USA) was used for data analysis. For normally distributed samples, Student’s *t*-test or one-way ANOVA variance was used to compare two or more groups, respectively. For non-normally distributed samples, either the Mann–Whitney test or the Kruskal–Wallis test was conducted to compare two or more groups, respectively. A post-hoc analysis with multiple comparisons revealed that the groups were significantly different from the others. Contingency analysis was used to evaluate categorical variables, whereas linear regression analysis was conducted to evaluate the correlation between variables. Statistical significance was set at *p* < 0.05. Receiver operating characteristic (ROC) curves were generated to identify the most discriminant values.

## 3. Results

### 3.1. Participant Selection and Eligibility

**Figure 1:**
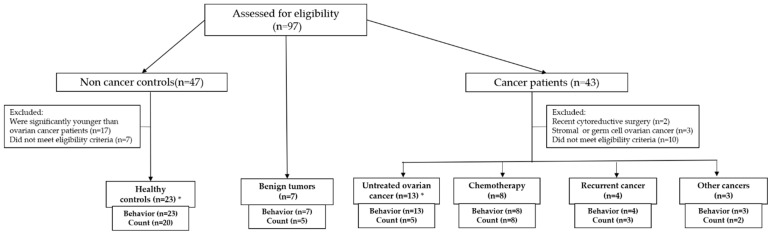
Schematic representation of this study's inclusion and exclusion criteria. Healthy participants were included based on age, lack of recent use of antiplatelet drugs, and ability to physically give blood samples. Patients with benign ovarian tumors or borderline diseases were included in the benign group. Ovarian cancer participants were included based on the diagnosis of epithelial ovarian cancer. Primary comparisons are indicated by asterisks. All other comparisons were exploratory. The bottom boxes show the numbers (n) of patient specimens evaluated using equal numbers of platelets (Behavior) and equal volumes of platelet-rich plasma (Number).

A total of 97 adults were assessed for eligibility to participate in this study (Figure 1). Among the participants, 23 healthy controls were eligible to participate based on age, overall health, medications, and no history of any cancer diagnosis (Healthy group). Seven patients had benign tumors or borderline diseases (four serous, one mucinous, one seromucinous, and one fibroid) that were not cancerous (Benign group). Thirteen newly diagnosed patients with ovarian cancer were chemotherapy and surgery naïve (Untreated group), eight patients with ovarian cancer were actively having or had chemotherapy in the past (Chemo group), four patients had recurrent ovarian cancer (Recurrent group), and three patients had other cancers including colon cancer (n = 1) and endometrial cancer (n = 2) that metastasized or invaded into the ovaries (Others group).

### 3.2. Demographics Were Similar among Participants, except for Race

General demographic data, including age, race, and ethnicity, were collected for all participants to evaluate the potential bias in the results from these factors (Table 1).

Participants were primarily of White race and non-Hispanic background, which is consistent with the demographics of patients with ovarian cancer in the catchment area of our cancer center. There were no significant differences in ethnicity, age, or BMI when compared across all groups. Among patients with ovarian cancer, there were no significant differences in cancer stage or histology. The levels of cancer antigen 125 (CA-125), an ovarian cancer biomarker, were significantly different among all the cancer groups. Untreated patients with ovarian cancer had slightly higher CA-125 levels whereas those receiving chemotherapy had lower CA-125 levels. However, a post-hoc analysis revealed no statistically significant differences in any individual cancer group when compared with all other cancer groups.

### 3.3. Elevated White Blood Cell and Platelet Counts in Untreated Were Reduced in Chemo

CBC analysis was used to compare blood parameters across the groups (Table 2). The following parameters were significantly different: white blood cells, neutrophils, hemoglobin, hematocrit, red blood cell width, and platelet count.

**Figure 2:**
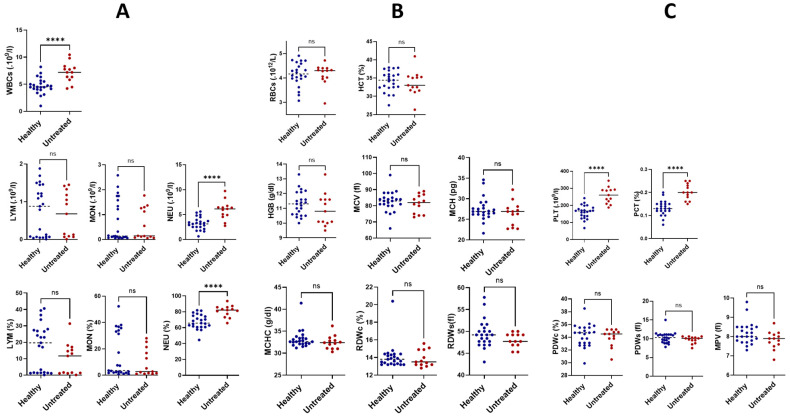
Comparison of CBCs in citrated whole blood between healthy controls and patients with untreated ovarian cancer. (**A**) white blood cells, (**B**) red blood cells, (**C**) platelets. Venous blood (8.5 mL) was collected using acid citrate dextrose (1.5 mL) anticoagulant. A VetScan HM5 Hematology Analyzer was used to count blood cells in each sample. (Healthy: Healthy controls, Untreated: patients with ovarian cancer before surgery or chemotherapy) Mann-Whitney or *t*-test; ns = not significant, **** *p* < 0.0001.

The difference in white blood cell counts can be attributed to the high neutrophil count in untreated patients with ovarian cancer, as neutrophils were the only white blood cell type evaluated that was significantly different among the groups. In addition, neutrophils are the most abundant white blood cells present in blood. The Chemo group had significantly lower hemoglobin and hematocrit values and lower red blood cell width compared to the other groups. Platelet count was the most significantly different CBC parameter between individual groups and the only CBC parameter that was significantly higher in all patients with cancer, including ovarian cancer (Untreated) and other cancers (Other), except for those receiving chemotherapy (Chemo). No significant differences were observed in other blood cell or platelet parameters, including size and platelet distribution width, across the groups. Plots of the individual patient CBC distributions for Untreated and Healthy groups show the profiles of significantly elevated white blood cells and neutrophils (Figure 2A), no differences in the red blood cells or parameters (Figure 2B), and significantly higher platelet counts and platecrit (Figure 2C) in patients with untreated ovarian cancer. There were no significant differences in platelet size or platelet distribution width between the two groups (Figure 2C). The platelet-to-lymphocyte ratio, which has been used to predict the risk of venous thromboembolism or patients with ovarian cancer prognosis [16,17], was derived from these data and found not to be significantly different between the Healthy and Untreated groups (*t*-test, ns).

### 3.4. Both Cancer-Altered Platelet Behavior and Numbers Are Associated with Increased Platelet Aggregation

**Figure 3:**
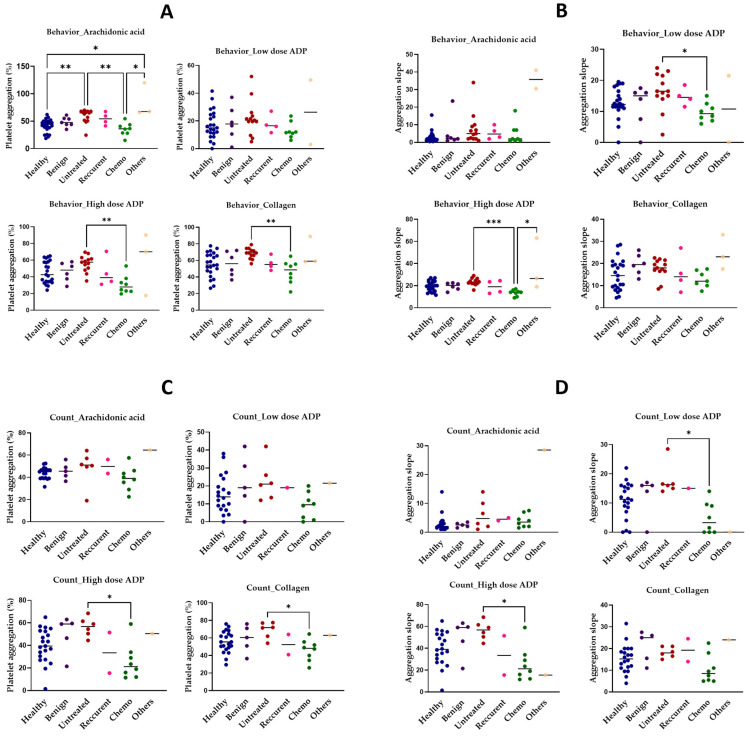
Platelet aggregation and aggregation slope comparing platelet behavior and platelet count. (**A**) Platelet aggregation comparing platelet behavior, (**B**) aggregation slope comparing platelet behavior, (**C**) platelet aggregation comparing platelet count, (**D**) aggregation slope comparing platelet count: Arachidonic acid, ADP, or collagen platelet aggregation agonists were used to induce platelet. Kruskal-Wallis test * *p* < 0.05, ** *p* < 0.01, *** *p* < 0.001.

To understand the potential impact of platelet count differences on platelet function in the different groups, we compared platelet aggregation parameters across the groups (Figure 3). Light transmission aggregometry measurement of platelet aggregation is a historical gold standard method for evaluating platelet physiology and diagnosing different platelet disorders when an appropriate platelet agonist is used [18]. There are multiple known potent commercially available platelet aggregation agonists, including: arachidonic acid, collagen, epinephrine, thrombin, and ristocetin. Arachidonic acid metabolites such as thromboxane A2 (TXA2) have also been shown to play a role in platelet and ovarian cancer crosstalk [19]. Once TXA2 binds to TXA2 receptors, TPα and TPβ, it activates platelets to release their granule contents in a positive feedback loop. Since only ADP and collagen have previously been studied as agonists of ovarian cancer-associated platelet aggregation and we did not know the driving cause of increased aggregation of platelets in patients with ovarian cancer, we tested multiple platelet agonists that target different parts of the aggregation pathway: arachidonic acid (AA: 0.5 mg/mL), collagen (Col: 0.19 mg/mL), and adenosine diphosphate (ADP: 20 µM/high-dose ADP). Low-dose ADP (ADP: 2 µM) was also tested to evaluate platelet hyper-activation. To evaluate the role of platelet behavior in platelet aggregation, specimen aliquots containing equal platelet numbers were used in the assay. Plots of aggregation versus time were used to determine platelet aggregation and the speed or rate of aggregation (aggregation slope) for each specimen. Significant differences were observed in platelet aggregation between the Healthy and Untreated or Others groups and between the Chemo and Untreated or Others groups for the AA (Figure 3A). This pattern was similar, but not statistically significant, for the other agonists, except that platelets in the Chemo group had significantly reduced platelet aggregation compared with the Untreated group for high-dose ADP and collagen.

The only significant difference observed for the aggregation slope when comparing platelet behavior across groups was the significantly lower rates for the Chemo group compared to the Untreated group for low-dose ADP and when compared to the Untreated or Other groups for high-dose ADP (Figure 3B). We also evaluated platelet aggregation using equal volumes of PRP to compare the impact of inherently different platelet counts in the various groups. The only significant differences observed when comparing platelet numbers in this way were between the Chemo and Untreated groups, with the Chemo group having significantly lower platelet aggregation when high-dose ADP and collagen agonists were used (Figure 3C) and aggregation slope when low- and high-dose ADP agonists were used (Figure 3D). A direct comparison of the platelet aggregation parameters between the Untreated and Healthy groups confirmed in more detail that the altered platelet aggregation in PRP from patients with ovarian cancer is mediated by both higher platelet counts and altered behavior. This was observed for all agonists tested in at least one of the four experimental conditions, and only consistently across all experimental conditions for high-dose ADP and for AA in three of the four experimental conditions (Table 3). Collagen-induced platelet aggregation, but not aggregation slope, was higher in the Untreated compared to the Healthy group when either equal platelet numbers or PRP volume was used.

ROC curves were generated to identify which endpoint or inducer exhibited the greatest discrimination between patients with ovarian cancer and Healthy controls (Table 4). Both platelet count and AA-induced aggregation exhibited area under the curve (AUC) values of 0.700, with platelet count exhibiting a *p*-value of 0.018 and AA-induced aggregation exhibiting a *p*-value of 0.021.

### 3.5. Platelet Count and PRP Volume Correlated with High-Dose ADP-Induced Platelet Aggregation in Patients with Cancer

Associations between platelet count or behavior with platelet aggregation or slope for each agonist were evaluated using linear regression analysis. There were two significant correlations between platelet count and platelet aggregation involving the AA agonist in the Healthy group and the high-dose ADP in the Untreated group (Table 5A) and one significant association between platelet behavior and aggregation slope for the AA agonist in the Untreated group (Table 5B). Platelet behavior was significantly correlated with platelet aggregation for the collagen agonists in the Healthy and Untreated groups and for the high-dose ADP and collagen agonists in the Untreated group (Table 5C). High-dose ADP was the only agonist associated with aggregation slope in the Healthy group while both low- and high-dose ADP were associated with aggregation slope in the Untreated group (Table 5D).

### 3.6. Patients with Ovarian Cancer Had Elevated P-Selectin and Beta-TG, but Not PF4

**Figure 4:**
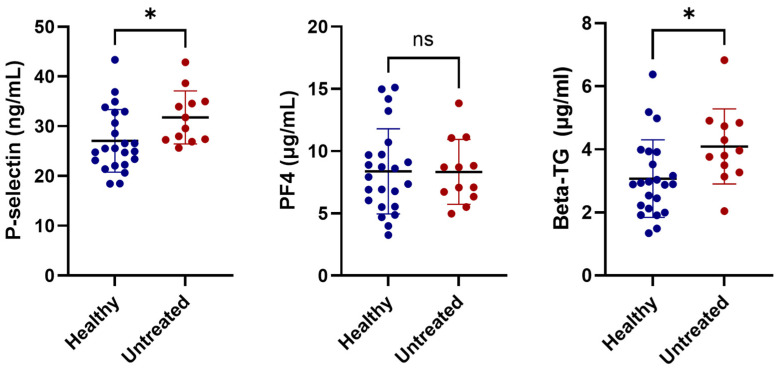
Concentrations of *P*-selectin, PF4, and beta-TG in PRP samples from healthy controls and patients with untreated ovarian cancer. *t*-test; ns = not significant, * *p* < 0.05.

Circulating platelet markers, including soluble P-selectin, PF4, and beta-TG, are commonly used to evaluate platelet activation. We used a multiplex ELISA assay to measure these markers in plasma collected from healthy controls and patients with untreated ovarian cancer to study the contribution of platelet pre-activation to the observed elevated platelet aggregation. Compared to plasma from healthy controls, plasma from patients with untreated ovarian cancer had elevated P-selectin (*p* = 0.03) and beta-TG (*p* = 0.02) levels, while PF4 levels were the same (*p* = 0.96) (Figure 4). Patients with ovarian cancer undergoing chemotherapy had significantly reduced plasma P-selectin and PF4 in comparison to untreated and healthy patients and significantly reduced plasma beta-TG in comparison to patients with ovarian cancer (Supplementary Figure S1).

## 4. Discussion

The results of this study support the hypothesis that platelets collected from patients with ovarian cancer exhibit altered behavior, in addition to elevated platelet counts. We observed that platelets from patients with ovarian cancer exhibited significantly elevated AA-induced and high-dose ADP-induced percent platelet aggregation when all groups were compared using equal platelet numbers to evaluate differences in platelet behavior.

For AA-induced platelet aggregation, platelets from patients with untreated ovarian cancer exhibited a higher percent platelet aggregation than those from healthy controls and chemotherapy-treated patients with ovarian cancer. This enhanced response to the AA agonist in ovarian cancer specimens suggests that platelets have elevated platelet aggregation pathway components downstream of AA, such as cyclooxygenase-2 (COX-2), which results in greater generation of thromboxanes and other pro-aggregation factors [20]. Previous studies have shown that the COX-2 enzyme is constitutively expressed in ovarian cancer cells and platelets [21]. This could explain why patients with ovarian cancer have elevated levels of AA-induced platelet activation since AA relies on the COX-2 enzyme to form pro-aggregation metabolites, which are drivers of the successful platelet aggregation process. Other pathways, i.e., collagen and ADP, rely on agonist-specific receptors that are only activated in the presence of an agonist.

For high-dose ADP-induced platelet aggregation, platelets from patients with untreated ovarian cancer exhibited greater aggregation only when compared to platelets from chemotherapy-treated patients with ovarian cancer. However, a direct comparison of platelets from patients with ovarian cancer and healthy controls revealed significantly increased platelet aggregation in patients with ovarian cancer with all agonists used at high-dose. This elevation was observed for both comparisons of equal numbers of platelets and equal volumes of PRP, suggesting that platelets from patients with ovarian cancer were pre-activated. Data in laryngeal carcinoma reported no differences in platelet count between high vs. low ADP-induced platelet aggregation cancer groups [22]. The aggregation slope (speed of aggregation) was also significantly higher for patients with ovarian cancer compared to healthy controls when low- or high-dose ADP was used, providing support for the concept that platelets in patients with ovarian cancer are both hyper-activated and pre-activated with respect to the rate of aggregation. The higher aggregation of ovarian cancer platelets might also be a result of increased ADP receptor availability on platelets or increased ADP signaling in patients with cancer. These results support targeting ADP receptors on platelets as a strategy to reduce the risk of thrombosis in patients with ovarian cancer. Another benefit of this approach could be the direct effect on the cancer itself, as ADP receptor targeting with drugs and genetic manipulation have been shown to reduce cancer burden [23].

The higher aggregation parameters in platelets observed in patients with untreated ovarian cancer compared to healthy controls suggest a cancer-associated change in platelet behavior, resulting in a higher risk of thrombosis. Among the possible changes that favor higher aggregation include the elevation of endogenous pro-aggregation factors, such as thrombin, Von Willebrand Factor (vWF), fibrinogen, and endogenous ADP, which amplify aggregation and formation of a stable platelet plug. It might also be a result of insufficient generation of anti-aggregation factors, such as antithrombins (ATI, ATII, and ATIII) or series-2 prostaglandins (PGD2, PGE2, and PGI2), which usually activate a negative feedback loop during the aggregation process to maintain normal hemostasis while preventing vessel blockage [20]. Previous research has shown elevated circulating calcium levels in patients with ovarian cancer pre-diagnosis [24], which could maintain stable platelet aggregates and increase aggregation. Although we did not evaluate coagulation in the current study, the formation of a stable platelet plug initiates the coagulation cascade, which recruits red blood cells to the site of the injury. In our study, we did not see differences in red blood cells; however, the hemoglobin and hematocrit levels were lower in patients with ovarian cancer undergoing chemotherapy. Reduction in hemoglobin and hematocrit levels are usually associated with anemia and increased bleeding risks, and this has been previously reported to be the case in ovarian and other patients with cancer undergoing chemotherapy [25].

Other platelet physical characteristics that could be contributing to an ovarian cancer-altered platelet behavior that further promotes ovarian cancer in a pathologic positive feed-forward loop have been reported in the literature [13,26,27,28]. Electron cryotomography has revealed shorter microtubules and elevated mitochondrial counts in platelets from patients with ovarian cancer [13]. The function of microtubules in resting platelets is to maintain a marginal band, which in turn preserves platelet shape [26]. During activation, microtubules support cytoskeletal reorganization by folding and supporting a star-like shape. Elevated mitochondrial levels drive platelet activation and support enhanced metabolism and energy production [29]. The combination of shorter microtubules and elevated mitochondria in platelets from patients with ovarian cancer implies that cancer platelets are more prone to activation due to less effort required for rearrangement of the cytoskeleton during activation and increased energy demands to support activation.

Lipids may also be involved in altering platelet behavior in patients with ovarian cancer. Analysis of the lipid composition of blood from patients with ovarian cancer revealed a preferential elevation of prothrombotic lysophospholipid and lower levels of antithrombotic lysophosphatidylcholine compared to healthy controls [27]. Mouse models of ovarian cancer and venous thrombosis have been used to demonstrate that cancer cell production of podoplanin, a membrane glycoprotein on their surface and on released extracellular vesicles, mediates both tumor growth and thrombosis [28]. Studies using an ovarian cancer organ-on-a-chip model have shown that platelets interact with tumor cells through glycoprotein VI and tumor galectin-3 under shear stress [30].

Ovarian cancer-altered platelets are likely to be affected by ascites (accumulation of fluid in the peritoneal cavity), a complication that arises with advanced ovarian cancer and occurs in more than 90% and 100% of patients with stage III and IV ovarian cancer, respectively [31]. Ascites form because of disrupted pressure forces between the extravascular and intravascular fluid spaces and could contribute to cancer-associated platelet alterations. Ascites fluid is rich in growth factors and have been reported to harbor platelets with activated fibrinogen receptors [32], indicating the presence of activated platelets in this fluid.

Patients with ovarian cancer have elevated levels of fibrinogen, which correlate with cancer stage and CA-125 levels, but not with age, BMI, and other coagulation indicators, such as prothrombin time, activated partial thromboplastin time, and thrombin time [33]. Elevated fibrinogen levels in patients with ovarian cancer have also been shown to be a single predictor of poor prognosis [34,35]. Patients with high circulating fibrinogen levels have reduced PFS and overall survival [34,35], indicating that fibrinogen might be another contributor to platelet hypercoagulability. High levels of other pro-coagulants such as vWF, D-dimer, tissue plasminogen activator antigen, and reduced ATIII have been associated with poor disease prognosis in ovarian cancer [35]. Survival analysis in laryngeal carcinoma showed that patients with higher levels of ADP-induced platelet aggregation had lower 5 years survival, further supporting the role of platelet aggregation in disease prognosis [22]. 

The physical parameters of platelets measured in this study suggest that platelet lifespan does not contribute to altered platelet behavior. The mean platelet volume and platelet distribution width were not significantly different across the groups in our study. The lack of a significantly higher number of smaller platelets in patients with cancer compared to healthy controls suggests that the elevated platelet count in these patients is not due to longer-lived platelets, which are characteristically smaller than younger platelets. The absence of significantly higher numbers of large platelets suggests that the elevated thrombosis capability of cancer-associated platelets is not due to pre-activated platelet states, because platelets increase in size upon activation and pre-activation [36]. Thus, although our functional aggregation assays indicate that ovarian cancer-associated platelets are pre-activated, their size profiles suggest that they may not be pre-activated, or that they are only partially pre-activated. Differences in platelet behavior might also be explained by changes that occur in platelet progenitor cells before the final platelets are produced. Platelet production in the bone marrow is regulated by paracrine signaling between interleukin-6 and thrombopoietin, which could contribute to increased platelet counts in patients with ovarian cancer [6].

Our results also confirm that altered platelet behavior, in addition to elevated platelet count, contributes to increased platelet aggregation in patients with ovarian cancer. A direct comparison of platelets from patients with ovarian cancer compared to healthy controls found a significantly increased percentage of platelet aggregation in patients with ovarian cancer with all agonists used at high doses. This elevation was observed for both comparisons of equal numbers of platelets and equal volumes of PRP, suggesting that platelets from patients with ovarian cancer were pre-activated. The aggregation slope was also significantly higher for patients with ovarian cancer than for healthy controls when low- or high-dose ADP was used, providing support for the concept that in patients with ovarian cancer, platelets are both hyper-activated and pre-activated with regard to the rate of aggregation.

Significantly elevated platelet counts were also observed in this study in patients with ovarian or other cancers that had metastasized to the ovary compared with healthy controls. This finding suggests that an elevated platelet count is not ovarian cancer-specific and can be generalized to other cancers, including colon and endometrial cancers. Previous studies have also shown an increased platelet count in ovarian cancer and other malignancies, as initially described by Trousseau [37] and others [6,14,38]. Elevated platelet count was also reported in other pelvic mass malignancies. Evaluation of adnexal tumors revealed that elevated platelet count in patients can be used to predict malignancy, and the addition of platelet count to existing biomarkers, such as CA-125, improved the predictive value of those biomarkers [39]. A meta-analysis of thrombocytosis in ovarian cancer suggests that thrombocytosis can be used as an independent prognostic factor for ovarian cancer, further supporting the importance of understanding platelets in ovarian cancer [40].

Previous data have shown that clear cell carcinoma ovarian cancer histology has a higher association with thrombosis risk than other ovarian cancer histologies [41,42,43,44]. In this study, there was an insufficient number of patients with clear cell carcinoma to identify differences in histology from other ovarian cancer histologies. Overall, there were no significant differences between platelet numbers or aggregation parameters based on disease stage, BMI, or cancer histology.

In this study, patients with recurrent ovarian cancer did not show any significant differences from the other groups, most likely because of the similarity of their blood cell properties to those of patients with untreated ovarian or other cancers. However, patients with ovarian cancer exposed to chemotherapy exhibited significantly reduced white blood cell and platelet counts compared to patients with untreated ovarian cancer, which is consistent with the known toxic effects of chemotherapy on bone marrow function and blood cells [25,45,46]. The lack of significance in neutrophil counts between untreated and chemotherapy-exposed patients with ovarian cancer suggests that chemotherapy toxicity to white blood cells is not specific to neutrophils. Multiple drugs currently used in cancer therapy reduce, or have the potential to reduce, platelet numbers in treated patients, including carboplatin, paclitaxel, pegylated liposomal doxorubicin, bevacizumab, dostarlimab, and niraparib. In the current study, platelets from patients exposed to chemotherapy exhibited lower rates of low-dose ADP-induced aggregation slope, but not percent aggregation, when compared to platelets from untreated patients. This suggests that chemotherapy causes platelet hypo-activation with respect to speed, but not total aggregation capacity. Chemotherapy-reduction of platelet numbers also contributes to the lower aggregation speed. The significant reduction in both aggregation speed and capacity of chemotherapy-exposed platelets compared to untreated platelets when high-dose ADP was used suggests that the platelets exposed to chemotherapy have reduced aggregation capacity and that the reduction of platelet numbers caused by chemotherapy also contributes to the reduced platelet aggregation in chemotherapy-treated patients.

Previous studies have reported inconsistent results regarding low-dose ADP hyper-activation of aggregation of platelets from patients with ovarian cancer compared to platelets from healthy and/or benign controls; however, both studies found no significant elevation in ovarian cancer platelets when high-dose ADP or collagen was used [13,14]. Our study evaluated both percent platelet aggregation and aggregation slope and observed significantly higher low-dose ADP induction of aggregation slope, but not percent platelet aggregation, in platelets from patients with ovarian cancer compared to healthy controls. Furthermore, our report expands upon this published research by including additional patient groups in the analysis (chemotherapy-treated versus newly diagnosed/untreated, and other cancers that have spread to the ovary) and evaluating platelet size as an indicator of platelet age, additional clinical endpoints, and platelet aggregation agonists. We found that patients with newly diagnosed ovarian cancer had elevated P-selectin and beta-TG levels, suggesting that platelets are pre-activated in patients with cancer. Our observation of elevated beta-TG levels in patients with untreated ovarian cancer and no change in PF4 levels is consistent with previous reports in patients with lung [47] and breast [48] cancers.

The elevated white blood cell counts observed in patients with ovarian cancer compared with healthy controls could be attributed to the elevated neutrophil counts in these patients. Patients with other cancers that had metastasized to the ovary also had higher neutrophil counts than the healthy controls; however, the number of patients was too low to achieve statistical significance. Although neutrophils can be a protective mechanism in normal immunology to trap and kill bacteria, they are associated with neutrophil extracellular traps, which worsen disease prognosis and increase the risk of thrombosis in patients with cancer [49,50]. For these reasons, an elevated neutrophil count, instead of all white blood cells, might be a better parameter for predicting thrombosis when using the Khorana score. Furthermore, the management of ovarian cancer should be personalized. In addition to thrombosis risk, increasing evidence suggests that the performance status of elderly patients should also be considered [51].

The use of platelet inhibitors in ovarian cancer and other cancers is a promising approach to prevent ovarian cancer and platelet crosstalk. Platelet inhibitors reduce tumor cell-induced platelet aggregation [52]. Accumulating evidence shows that different classes of platelet inhibitors have direct anticancer effects and enhance the efficacy of other anticancer drugs. COX inhibitors, such as aspirin, inhibited cancer cell migration and proliferation, and increased apoptosis and cisplatin sensitivity [53]. ADP receptor targeting drugs (cangrelor, clopidogrel, and ticagrelor) reduced tumor growth, increased paclitaxel sensitivity, and reversed platelet-induced angiogenesis and cancer cell cycle progression, apoptosis, and autophagy [23,54,55]. The ADP reuptake drug, dipyridamole, has been shown to overcome cisplatin resistance and increase etoposide, interferon, and tumor necrosis factor anticancer effects [56,57,58,59]. Additionally, vorapaxar, a protease-activated receptor-1 antagonist that prevents thrombin signaling, inhibits thrombin-induced ovarian cancer cell line proliferation [60]. In addition to platelet inhibitors, the use of anticoagulants including direct oral anticoagulants, vitamin K, and heparin-targeting drugs is recommended for the management of thrombosis in patients with cancer [61].

While the use of platelet inhibitors in ovarian cancer is an attractive approach to prevent thromboembolisms and treat cancer, precaution should be taken when using these drugs in patients with active cancer. We and others [25] have shown that chemotherapy induces an anemic phenotype in patients with cancer, which puts these patients at a high risk of deadly bleeding. Platelet inhibitors and anticoagulants also cause gastrointestinal toxicity [62], hence, these drugs should only be used when the benefits outweigh the risks. Some novel anticoagulants generally seem to be a better choice when it comes to anticoagulant use in patients with cancer as they do not impose as much bleeding risks on patients compared to vitamin K or heparin targeting drugs and they result in fewer drug–drug interactions [61,63].

A limitation of our study is the number of specimens evaluated; however, the findings are significant and justify larger studies to further evaluate the relationships between thrombosis and clinical endpoints, including tumor histology and patient demographics. Additional studies on the mechanism underlying platelet hyperactivation in patients with ovarian cancer will provide new insights that could be translated into thrombosis prevention strategies. Another limitation of our study is that we used a low number of platelets in the aggregation assay, which allowed for equal platelet numbers to be compared across all groups because the chemotherapy patients had very low platelet counts. Finally, the use of ACD as an anti-coagulant when collecting blood could have limited the comparison of our results with data generated in other laboratories that use ethylenediaminetetraacetic acid as an anticoagulant when collecting blood, however we have corrected for this in the calculations.

## 5. Conclusions

The findings of this study demonstrate that patients with ovarian cancer have significantly higher neutrophil and platelet counts and altered platelet behavior. AA and ADP were found to be involved in the mechanism of altered platelet behavior. This is the first report of elevated AA-induced platelet aggregation in patients with ovarian cancer. According to our findings, it appears that platelets are influenced by ovarian cancer in a way that results in not only an increase in their quantity but also an enhancement of their ability to aggregate. The increased platelet aggregation in turn releases the platelet content, which could increase cancer aggressiveness. Overall, these findings justify a larger and more detailed study to better understand cancer-associated hypercoagulability and to develop intervention strategies to reduce the risk of thrombosis and deaths caused by thrombosis in patients with ovarian cancer.

## Figures and Tables

**Table 1:** Participant demographics and cancer status.

Parameter		Healthy (n = 23)	Benign (n = 7)	Untreated (n = 13)	Recurrent (n = 4)	Chemo (n = 8)	Others (n = 3)	*p*-Value
Ethnicity								
	Non-Hispanic	20	7	13	4	6	2	0.43
	Hispanic	1	0	0	0	1	1	
	Unknown	2	0	0	0	1	0	
Race								
	White	22	6	13	3	7	2	<0.01
	Black	0	0	0	0	1	1	
	Asian	0	0	0	0	0	1	
	American Indian	0	0	0	1	0	0	
	Unknown	1	1	0	0	0	0	
Age	Median	59	57	60	57	58	53	0.93
BMI	Median	N/A	23.7	29.0	38.0	26.7	24.4	0.34
Cancer stage								
	I	N/A	N/A	3	0	0	N/A	0.25
	II	N/A	N/A	2	0	0	N/A	
	III	N/A	N/A	8	3	7	N/A	
	IV	N/A	N/A	0	1	1	N/A	
Cancer Histology								
	High-grade serous	N/A	N/A	6	2	5	N/A	0.64
	Low-grade serous	N/A	N/A	0	1	1	N/A	
	Clear cell	N/A	N/A	3	0	0	N/A	
	Endometrioid	N/A	N/A	1	0	1	N/A	
	Mucinous	N/A	N/A	1	1	0	N/A	
	Carcinosarcoma	N/A	N/A	1	0	1	N/A	
	Adenocarcinoma	N/A	N/A	1	0	0	N/A	
CA-125	Median	N/A	N/A	256.0	41.85	31.35	219.0	0.03

N/A: Not Available or Applicable.

**Table 2:** Comparison of CBC parameters across groups.

Parameter ^#^	Healthy (n = 23)	Benign (n = 7)	Untreated (n = 13)	Recurrent (n = 4)	Chemo (n = 8)	Others (n = 3)	*p*-Value
WBC (×10^9^/L)	5.294	6.106	8.459	8.212	5.394	13.471	<0.01
LYM (×10^9^/L)	1.035	0.094	0.800	0.865	0.082	0.676	0.42
MON (×10^9^/L)	0.165	1.859	0.176	0.894	0.882	0.688	0.20
NEU (×10^9^/L)	3.706	4.129	7.247	5.894	4.100	12.106	<0.001
RBC (×10^12^/L)	4.953	5.024	5.059	4.882	4.388	4.588	0.11
HGB (g/dL)	13.29	14.35	12.71	13.41	11.00	10.53	0.0001
HCT (%)	40.49	43.14	38.86	41.39	34.69	31.85	<0.001
MCV (fL)	97.65	98.82	96.47	98.24	95.29	82.94	0.85
MCH (pg)	31.65	31.06	31.65	32.00	31.00	27.41	0.77
MCHC (g/dL)	38.35	37.65	38.12	38.24	37.41	38.77	0.19
RDW (%)	16.24	16.24	15.88	17.77	17.88	18.24	<0.01
PLT (×10^9^/L)	196.47	196.47	305.88	277.06	134.12	468.82	<0.0001
MPV (fL)	9.412	9.765	9.294	9.412	8.882	8.941	0.12
PDW (%)	40.82	40.47	40.59	41.35	38.47	38.24	0.33

^#^ Parameters adjusted with a correction factor accounting for ACD dilution effect. Abbreviations: WBC: White blood cells, LYM: Lymphocytes, MON: Monocytes, NEU: Neutrophils, RBC: Red blood cells, HGB: Hemoglobin, HCT: Hematocrit, MCV: Mean corpuscular volume, MCH: Mean corpuscular hemoglobin, MCHC: Mean corpuscular hemoglobin concentration, RDW: Red blood cell distribution width, PLT: Platelets, MPV: Mean platelet volume, PDW: Platelet distribution width.

**Table 3:** Platelet aggregation and aggregation slope with platelet agonists. A: Platelet aggregation comparing platelet behavior. B: Aggregation slope comparing platelet behavior. C: Platelet aggregation comparing numbers. D: Aggregation slope comparing platelet count in untreated cancer: Patients with untreated ovarian cancer before intervention with surgery or chemotherapy. Statistical significance was set at *p* < 0.05. (Healthy: Healthy controls, Untreated: Patients with ovarian cancer before surgery or chemotherapy).

**A**				**B**			
**10^7^** **Platelets/** **Reaction**	**Healthy**	**Untreated**	***p*-Value**	**10^7^** **Platelets/** **Reaction**	**Healthy**	**Untreated**	***p*-Value**
Arachidonic Acid (0.5 mg/mL)	44.00	65.00	<0.0001	Arachidonic Acid (0.5 mg/mL)	02.00	05.00	<0.01
ADP (2 µM)	15.00	20.00	0.28	ADP (2 µM)	12.25	16.25	0.03
ADP (20 µM)	42.50	57.50	0.03	ADP (20 µM)	19.50	22.50	0.02
Collagen (0.19 mg/mL)	54.50	69.00	<0.01	Collagen (0.19 mg/mL)	15.25	17.75	0.19
**C**				**D**			
**25 µL PRP/** **Reaction**	**Healthy**	**Untreated**	***p*** **-Value**	**25 µL PRP/** **Reaction**	**Healthy**	**Untreated**	***p*-Value**
Arachidonic Acid (0.5 mg/mL)	45.00	51.00	0.04	Arachidonic Acid (0.5 mg/mL)	02.00	04.75	0.21
ADP (2 µM)	13.90	21.00	0.14	ADP (2 µM)	11.25	16.25	0.02
ADP (20 µM)	39.50	56.75	<0.01	ADP (20 µM)	14.75	22.00	<0.01
Collagen (0.19 mg/mL)	55.50	71.75	0.02	Collagen (0.19 mg/mL)	15.25	18.00	0.14

**Table 4:** Summary of ROC values comparing patients with ovarian cancer and healthy controls.

Parameter	Behavior		Number	
	AUC	*p*-Value	AUC	*p*-Value
Platelet Count	0.70	0.02		
AA	0.70	0.02	0.52	0.83
ADP (2 µM)	0.51	0.90	0.51	0.96
ADP (20 µM)	0.51	0.91	0.53	0.79
Col	0.59	0.27	0.51	0.96

**Table 5:** Linear regression analysis of platelet aggregation association with platelet count and behavior.

**A**			**B**		
**10^7^** **Platelets/** **Reaction**	**Healthy**	**Untreated**	**10^7^** **Platelets/** **Reaction**	**Healthy**	**Untreated**
Arachidonic Acid (0.5 mg/mL)	R^2^ = 0.19 *p* = 0.04	R^2^ = 0.04 *p* = 0.51	Arachidonic Acid (0.5 mg/mL)	R^2^ = 0.00 *p* = 0.95	R^2^ = 0.40 *p* = 0.02
ADP (2 µM)	R^2^ < 0.01 *p* = 0.74	R^2^ = 0.31 *p* = 0.06	ADP (2 µM)	R^2^ = 0.02 *p* = 0.57	R^2^ = 0.09 *p* = 0.35
ADP (20 µM)	R^2^ = 0.00 *p* = 0.98	R^2^ = 0.38 *p* = 0.02	ADP (20 µM)	R^2^ = 0.09 *p* = 0.16	R^2^ = 0.00 *p* = 0.99
Collagen (0.19 mg/mL)	R^2^ = 0.15 *p* = 0.072	R^2^ = 0.30 *p* = 0.60	Collagen (0.19 mg/mL)	R^2^ = 0.03 *p* = 0.38	R^2^ = 0.13 *p* = 0.23
**C**			**D**		
**25 µL PRP/** **Reaction**	**Healthy**	**Untreated**	**25 µL PRP/** **Reaction**	**Healthy**	**Untreated**
Arachidonic Acid (0.5 mg/mL)	R^2^ = 0.05 *p* = 0.35	R^2^ = 0.00 *p* = 0.99	Arachidonic Acid (0.5 mg/mL)	R^2^ = 0.04 *p* = 0.38	R^2^ = 0.18 *p* = 0.40
ADP (2 µM)	R^2^ = 0.05 *p* = 0.38	R^2^ = 0.43 *p* = 0.15	ADP (2 µM)	R^2^ = 0.10 *p* = 0.20	R^2^ = 0.70 *p* = 0.04
ADP (20 µM)	R^2^ = 0.07 *p* = 0.26	R^2^ = 0.75 *p* = 0.03	ADP (20 µM)	R^2^ = 0.44 *p* < 0.01	R^2^ = 0.93 *p* < 0.01
Collagen (0.19 mg/mL)	R^2^ = 0.21 *p* = 0.04	R^2^ = 0.73 *p* = 0.03	Collagen (0.19 mg/mL)	R^2^ = 0.14 *p* = 0.10	R^2^ = 0.01 *p* = 0.92

## Data Availability

The original contributions presented in this study are included in the article, further inquiries can be directed to the corresponding author.

## Supplementary Materials

The following supporting information can be downloaded at: https://www.mdpi.com/article/10.3390/cancers16213583/s1, Figure S1: Concentrations of P-selectin, PF4, and beta-TG in PRP samples from Healthy, Untreated and Chemo groups.

## Abbreviations

Abbreviation	Full Name
AA	arachidonic acid
ACD	Acid citrate dextrose
ADP	Adenosine diphosphate
AT	Antithrombin
AUC	Area under the curve
beta-TG	β-thromboglobulin
BMI	Body mass index
CA-125	Cancer antigen 125
CBC	Complete blood count
Col	collagen
COX	Cyclooxygenase
HCT	Hematocrit
HGB	Hemoglobin
IL-6	Interleukin-6
IRB	Institutional Review Board
LYM	Lymphocytes
MCH	Mean corpuscular hemoglobin
MCHC	Mean corpuscular hemoglobin concentration
MCV	Mean corpuscular volume
MON	Monocytes
MPV	Mean platelet volume
NEU	Neutrophils
PAP	Platelet Aggregometer Profiler
PDW	Platelet distribution width
PF4	Platelet factor 4
PFS	Progression-free survival
PG	Prostaglandin
PLT	Platelets
PPP	Platelet poor plasma
PRP	Platelet-rich plasma
RBC	Red blood cells
RDW	Red blood cell distribution width
ROC	Receiver operating characteristic
TP	Thromboxane receptor
TXA2	Thromboxane A2
vWF	Von Willebrand Factor
WBC	White blood cells
