# Supplementary material for: Elevated Platelet Aggregation in Patients with Ovarian Cancer: More than Just Increased Platelet Count
Source: Cancers (Basel). 2024 Oct 24;16(21):3583. doi: 10.3390/cancers16213583 (PMC11545395; doi:10.3390/cancers16213583)

**A**

**B**

**C**

**Supplementary Figure S1.** Concentrations of P-selectin, PF4, and beta-TG in PRP samples from Healthy, Untreated and Chemo groups. (**A**). P-Selectin, (**B**). PF4, (**C**). Beta-TG, ANOVA, ns: not significant, *p<0.05, **p<0.01, ***p<0.001.


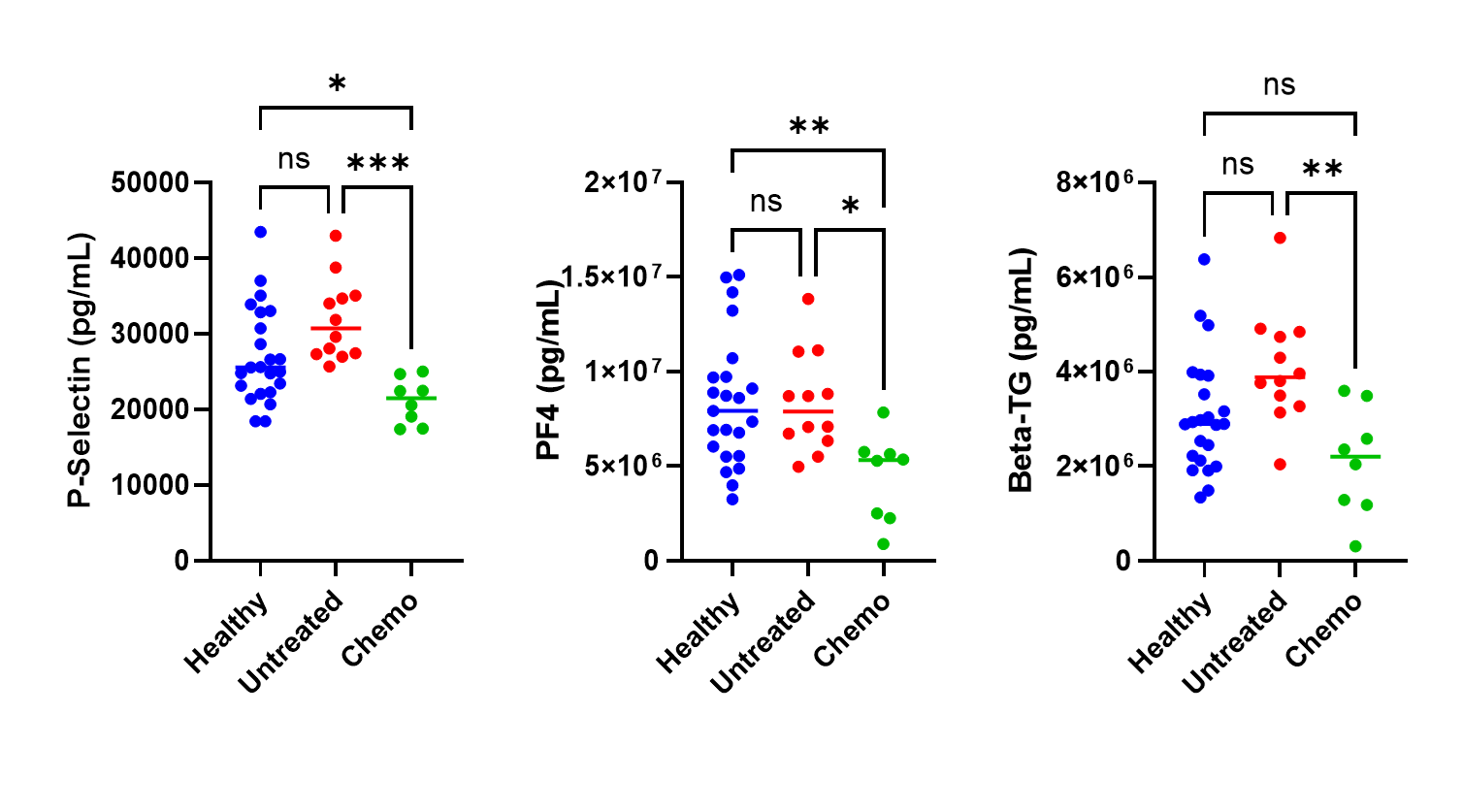

Supplement: Supplementary file 1 [file cancers-16-03583-s001.zip › cancers-3247574-supplementary.docx]
